# Supplementary material for: Estimating climate change and mental health impacts in Canada: A cross-sectional survey protocol
Source: PLoS One. 2023 Oct 11;18(10):e0291303. doi: 10.1371/journal.pone.0291303 (PMC10566728; doi:10.1371/journal.pone.0291303)
Supplement: S1 Table — (DOCX) [file pone.0291303.s001.docx]

# S1. Appendix 1. Questionnaire

## Notes:

- **Formatting:** The questionnaire was administered using software; therefore, the formatting in this appendix does not represent how the questionnaire visually looked for participants.
- **[Square Brackets]:** Text within [square brackets] are notes for the interviewer and/or coding for analyses that will not be seen/heard by participants
- **Climate Change:** If needed by the participant in the survey, the following definition of “climate change” will act as a prompt or example:
  - Climate change includes changes in temperature, precipitation, wind patterns, or other effects that occur over several decades. Climate change also includes climate hazards, including, but not limited to, extreme events such as a heatwave, wildfire, flood, drought, and severe storm, as well as sea-ice loss, thawing permafrost, and changing ecosystems.

## Questionnaire:

1. How old are you?

*[open text (numerical) field]*

- - Specify: _____ years old [skip to Q3]
  - [Prefer not to answer]* [proceed to Q2]

1. [*If participant does not want to answer, then interviewer will prompt]*

We understand your hesitation to provide your age. This information is needed to participate in the survey. Would you feel comfortable confirming which age category you fall under? Are you:

*[select one]*

- - 13 to 15 years old
  - 16 to 18 years old
  - 19 to 24 years old
  - 25 to 29 years old
  - 30 to 34 years old
  - 35 to 39 years old
  - 40 to 44 years old
  - 45 to 49 years old
  - 50 to 54 years old
  - 55 to 59 years old
  - 60 to 64 years old
  - 65 to 69 years old
  - 70 to 74 years old
  - 75 to 79 years old
  - 80 years old or older
  - [Prefer not to answer] [*If participant still refuses to answer, then survey is terminated]*

Please rate your level of agreement with the following statements.

*[select one for each row]*

|  | Strongly disagree | Disagree | Neither agree nor disagree | Agree | Strongly agree | [Prefer not to answer] |
| --- | --- | --- | --- | --- | --- | --- |
| 1. I have been directly affected by climate change or a climate hazard, such as a heatwave, wildfire, flood, drought and so on |  |  |  |  |  |  |
| 1. I know someone who has been affected by climate change |  |  |  |  |  |  |

How much do you feel each of the following emotions when you think about climate change?

*[select one for each row]*

|  | Not at all | A little | A moderate amount | A lot | Extremely | [Prefer not to answer] |
| --- | --- | --- | --- | --- | --- | --- |
| 1. Sad |  |  |  |  |  |  |
| 1. Angry |  |  |  |  |  |  |
| 1. Grief |  |  |  |  |  |  |
| 1. Helpless |  |  |  |  |  |  |
| 1. Hopeless |  |  |  |  |  |  |
| 1. Worried |  |  |  |  |  |  |
| 1. Anxious |  |  |  |  |  |  |
| 1. Depressed |  |  |  |  |  |  |
| 1. Tense |  |  |  |  |  |  |
| 1. Concerned |  |  |  |  |  |  |
| 1. Stressed |  |  |  |  |  |  |
| 1. Scared |  |  |  |  |  |  |
| 1. Powerless |  |  |  |  |  |  |

Please rate how much each of the following statements are true for you.

*[select one for each row]*

|  | Not at all | A little | A moderate amount | A lot | Extremely | [Prefer not to answer] |
| --- | --- | --- | --- | --- | --- | --- |
| 1. The way I feel about climate change is because of *past and current* climate change impacts |  |  |  |  |  |  |
| 1. The way I feel about climate change is because of *anticipated future* climate change impacts |  |  |  |  |  |  |
| 1. The way I feel about climate change is because of things that I see or read about in the news or on social media |  |  |  |  |  |  |

Please indicate the extent to which each of the following statements reflects your own response to climate change.

*[select one for each row]*

|  | Strongly disagree | Disagree | Neither agree nor disagree | Agree | Strongly agree | [Prefer not to answer] |
| --- | --- | --- | --- | --- | --- | --- |
| 1. The threat of climate change is affecting my quality of life |  |  |  |  |  |  |
| 1. The more I learn about climate change, the more anxious I become |  |  |  |  |  |  |
| 1. It saddens me to think that we may lose particular areas and species because of climate change |  |  |  |  |  |  |
| 1. I experience some distress when I think about the uncertainties and unknowns associated with climate change |  |  |  |  |  |  |
| 1. At times, I feel overwhelmed when thinking about the uncertain, unpredictable, and uncontrollable future impact of climate change |  |  |  |  |  |  |

Please rate how often the following statements are true for you.

*[select one for each row]*

|  | Never | Rarely | Sometimes | Often | Almost Always | [Prefer not to answer] |
| --- | --- | --- | --- | --- | --- | --- |
| 1. Thinking about climate change makes it difficult for me to concentrate. |  |  |  |  |  |  |
| 1. Thinking about climate change makes it difficult for me to sleep. |  |  |  |  |  |  |
| 1. I have nightmares about climate change. |  |  |  |  |  |  |
| 1. I find myself crying because of climate change. |  |  |  |  |  |  |
| 1. I think, “why can’t I handle climate change better?” |  |  |  |  |  |  |
| 1. I go away by myself and think about why I feel this way about climate change. |  |  |  |  |  |  |
| 1. I write down my thoughts about climate change and analyze them. |  |  |  |  |  |  |
| 1. I think, “why do I react to climate change this way?” |  |  |  |  |  |  |
| 1. My feelings about climate change negatively affect my daily life, which includes at least one of the following: eating, concentrating, work, school, sleeping, spending time in nature, playing, having fun, or relationships. |  |  |  |  |  |  |

Please rate how often each of the following statements are true for you.

*[select one for each row]*

|  | Never | Rarely | Sometimes | Often | Almost always | [Prefer not to answer] |
| --- | --- | --- | --- | --- | --- | --- |
| 1. I use products that are more environmentally friendly |  |  |  |  |  |  |
| 1. To address climate change, when possible, I use products that are made locally |  |  |  |  |  |  |
| 1. To address climate change, I make changes to my diet, such as incorporating vegetarian or vegan options, seasonal produce, or consuming less meat |  |  |  |  |  |  |
| 1. I join climate-related protests or marches |  |  |  |  |  |  |
| 1. I consider changing who I vote for because of climate change concerns^[[1]](#footnote-1)^ |  |  |  |  |  |  |
| 1. I express my views about climate change openly to friends and family or on social media |  |  |  |  |  |  |

1. [Telephone version] What is your current gender identity?

*[DO NOT READ LIST]*

*[*For the online survey, this question will be replaced with “Do you identify as… (choose all that apply)]*

- - Man
  - Woman
  - Two-Spirit^[[2]](#footnote-2)^
  - Gender fluid^[[3]](#footnote-3)^
  - Non-binary^[[4]](#footnote-4)^
  - Another gender identity (please specify) ­­­­­­­__________
  - [Prefer not to answer]

1. With which ethnic background or backgrounds do you identify?
   - Arab
   - Black
   - Chinese
   - Filipino
   - Japanese
   - Indigenous (including Inuit, First Nations, Métis)
   - Korean
   - Latin American
   - South Asian (e.g., East Indian, Pakistani, Sri Lankan, etc.)
   - Southeast Asian (including Vietnamese, Cambodian, Laotian, Thai, etc.)
   - West Asian (e.g., Iranian, Afghan, etc.)
   - White
   - Population group not listed above. I identify as ____________ (option to specify)
   - [Prefer not to answer]
2. What is your postal code?

*[open text]*

- - Specify (enter 6-digit postal code): ________
  - [Prefer not to answer]

1. Please give your best estimate of your total income in the past 12 months. Was it:

*[If participant is hesitant to provide this information, interviewer prompt: There are important relationships between health and income. Please be assured that, like all other information you have provided, your answer will be anonymous*.] *[Note: income includes received by all household members, from all sources, before taxes and deductions]*

*[select one]*

- - Less than $30,000
  - $30,000 or more, but under $60,000
  - $60,000 or more, but under $80,000
  - $80,000 or more, but under $100,000
  - $100,000 or more
  - [Unsure]
  - [Prefer not to answer]

That is all of the questions we have. Thank you for taking the time to complete this survey.

1. This item is only displayed if Q1/Q2 ≥ 18 years of age [↑](#footnote-ref-1)
2. The following information will only be read if the participant asks for clarification: Two-Spirit is an umbrella term used by some Indigenous Peoples from Turtle Island/North America to describe their sexual, gender, and/or spiritual identity. [↑](#footnote-ref-2)
3. The following information will only be read if the participant asks for clarification: Gender Fluid refers to a gender identity that is changing or “fluid” [↑](#footnote-ref-3)
4. The following information will only be read if the participant asks for clarification: Nonbinary (also Non-Binary) is a preferred umbrella term for all genders other than female/male or woman/man. [↑](#footnote-ref-4)
